# Assessment of psychosocial risks in Electricity and Gaz Company

**DOI:** 10.1192/j.eurpsy.2023.2038

**Published:** 2023-07-19

**Authors:** A. Hrairi, N. Kammoun, N. Rmadi, R. Masmoudi, K. Jmal Hammami, M. L. Masmoudi, J. Masmoudi, M. Hajjaji

**Affiliations:** 1 Occupational medicine, University of Sfax; 2 Tunisian Health and Safety Institute, Tunis; 3 Occupational medicine; 4 Psychiatry A department, Hedi Chaker Hospital Sfax, Sfax, Tunisia

## Abstract

**Introduction:**

Risk evaluation is a global process covering different aspects of employee’s workandfamily life. Nowadays, psychosocial risks are as important as physical and chemical risks, and their identification isdeterminant in each workplace.

**Objectives:**

Our study aimed to assess psychosocial risks among Electricity and Gas Company’s employees and to identify factors related to these risks.

**Methods:**

A cross sectional study was conducted among male workers in a Tunisian electricity and gas Company. The KRASEK scale was used to assess psychosocial risks. The Statistical analysis was performed with SPSS version 23.

**Results:**

Among male workers in the company, 83 employees participated in this study. The mean age of our population was 41.28 years± 12.12 years. Manuel labour was identified in 67.5% of cases. High psychological demands were reported by 63.9% of the employees. The assessment of decision latitude dimension identified low autonomy at work in 54.2% of cases. The mean social support scale was 23.73± 4.18. Job strain was identified among 32.5% of participants. Among employees in job strain, twenty-one subjects (77.8%) were affected in the technical division and 21.7% were in “iso strain”. Job strain and iso strain were associated with sedentary workers aged less than 45 years, p values were 0.006 (OR= 5.474; IC 95% [1.477-20.290]) and 0.010 (OR= 4.917; IC 95% [1.353-17.872]) respectively. However, Iso strain was negatively associated with being married (p=0.038) (0.0327 IC 95% [0.111-0.964]).

**Conclusions:**

This study highlighted the importance of psychosocial risks in this company. The identification of these risks in the workplace may further help preventers to recommend proper interventions to offer employees a supportive work environment and to enhance their personal and professional well-being.

**Disclosure of Interest:**

None Declared

